# Enhanced Glucose Recovery from Reed Straw (*Phragmites australis*) via Cascade Microwave–Alkali and Ultrasound–H_2_O_2_ Pretreatment with Sophorolipid/BSA Supplementation

**DOI:** 10.3390/polym18182284

**Published:** 2026-09-18

**Authors:** Yaru Xiong, Lei Wang, Qingqing Zhou, Fangcai Li, Fangling He, Rongjiao Liu, Juan Wang, Zhifei Zhan, Huayun Jia, Chao Xu, Liang Cai

**Affiliations:** 1 Hunan Provincial Center for Disease Control and Prevention, Changsha 410005, China; 2 School of Food and Bioengineering, Changsha University of Science and Technology, Changsha 410005, China

**Keywords:** lignocellulosic biomass, reed straw (*Phragmites australis*), pretreatment, microwave–alkali, ultrasound–H_2_O_2_, enzymatic hydrolysis

## Abstract

The growing demand for renewable fuels has led to lignocellulosic biomass being turned to as a feedstock for fermentable sugars, yet its hierarchical structure severely limits enzymatic saccharification. In this study, a cascade of microwave-assisted alkali and ultrasound-assisted H_2_O_2_ pretreatment, combined with sophorolipid and BSA supplementation, was established to enhance glucose recovery from reed straw under mild conditions while substantially reducing enzyme and chemical inputs. The cascade pretreatment selectively removed lignin and hemicellulose, increasing the apparent cellulose crystallinity index from 58.77% to 69.16%. This apparent increase is attributed to the removal of non-cellulosic components rather than to enhanced cellulose crystallinity. This structural modification boosted glucose yield by 1.28-fold over single-stage alkali pretreatment and 6.29-fold over raw material. Supplementation with β-glucosidase, sophorolipid, and BSA further enhanced the yield by 20.34% at a reduced cellulase loading of 12 FPU/g. Fractal kinetic analysis showed that the combined addition increased the rate constant from 0.0067 to 0.0111 while reducing the fractal dimension from 0.7136 to 0.6787. Fed-batch hydrolysis at 25% solids achieved 128.71 g/L glucose, recovering 31.22 g per 100 g raw material. This integrated strategy offers a viable route for converting reed straw into high-titer fermentable sugars under mild conditions with substantially reduced enzyme and chemical inputs.

## 1. Introduction

The global transition toward carbon neutrality has intensified the search for scalable routes to convert renewable biomass into fuels and chemicals. Among various lignocellulosic feedstocks, the common reed (*Phragmites australis*) is particularly abundant in China, covering approximately 1.3 million hectares and yielding over 2.2 million tons annually [1]. However, the decline of traditional applications such as papermaking has left vast quantities of reed straw underutilized, resulting in both resource wastage and environmental concerns about residue accumulation [2]. Developing an efficient biorefinery platform for this abundant feedstock is therefore of both economic and ecological significance.

A major hurdle in converting lignocellulosic biomass is the physical and chemical shielding of cellulose microfibrils by the surrounding lignin–hemicellulose polymer network. Alkaline pretreatment can disrupt this barrier through saponification of ester linkages in the lignin–carbohydrate complex (LCC), yet it typically requires high alkali dosages and prolonged residence times and generates inhibitory byproducts. Hydrogen peroxide addition enhances delignification via oxidative cleavage of lignin β-O-4 ether bonds but introduces safety concerns due to gas evolution under pressurized conditions [3,4]. Physical-field assistance (microwave and ultrasound) can accelerate mass transfer and improve pretreatment efficacy under milder conditions; however, each approach has inherent limitations when used alone—microwave treatment suffers from a heterogeneous energy distribution, while ultrasound has limited penetration depth in dense lignocellulosic matrices [5,6]. This motivates a cascade design that harnesses the complementary strengths of both technologies to achieve stepwise deconstruction of the polymer composite.

On the saccharification side, cellulase performance is critically constrained by two phenomena rooted in polymer interfacial chemistry: (i) non-productive enzyme adsorption onto lignin surfaces via hydrophobic and electrostatic interactions and (ii) mass-transfer limitations arising from high system viscosity under high-solids operation (>15% solids) [7]. Although additives such as biosurfactants and non-catalytic proteins can mitigate these issues, their synergistic mechanisms, particularly in modulating the polymer–enzyme interfacial interactions and alleviating diffusional constraints, remain poorly understood, and quantitative kinetic descriptions are lacking [8,9]. Furthermore, while fed-batch feeding enables high-solids processing, its operational feasibility and maximum achievable sugar concentrations have not been systematically evaluated for reed straw.

To address these knowledge gaps, the specific objectives of this study were to: (i) develop a mild cascade pretreatment (80 °C, ambient pressure) combining microwave-assisted alkaline treatment and ultrasound-assisted H_2_O_2_ oxidation for systematic deconstruction of the lignocellulosic polymer complex and to characterize the structural evolution (composition, crystallinity, and porosity) underlying the enhanced enzymatic accessibility; (ii) construct a low-cellulase saccharification system supplemented with sophorolipid and BSA as dual-functional additives and quantitatively decouple their synergistic mechanisms using fractal kinetic analysis; and (iii) establish a fed-batch high-solids hydrolysis regime at 25% (*w*/*v*) loading to achieve industrially relevant glucose titers (>100 g/L). Through this integrated platform, we aim to reduce chemical and enzyme inputs while providing a practical route for reed straw valorization.

## 2. Materials and Methods

### 2.1. Raw Materials and Chemical Reagents

Reed straw was collected from the shores of Dongting Lake, Hunan Province, China. Prior to use, the air-dried reed straw was first chipped into small sections (3–5 cm) using a laboratory-scale chipper and then finely ground in a high-speed universal crusher (FW-100; Taisite Instrument Co., Ltd., Tianjin, China) equipped with a 2 mm stainless steel mesh sieve. The resulting powder passing through the sieve was collected without further size fractionation (particle size distribution: fine dust to ~2 mm, with a minor fraction up to approximately 2.5 mm) and dried at 60 °C until a constant weight was achieved. The cellulase Cellic^®^ CTec3 (150 FPU/mL) and β-glucosidase Viscozyme L (100 FBG/g) were supplied by Novozymes (Tianjin, China) Biotechnology Co., Ltd. Sophorolipid (purity of 99%) was purchased from Pioneer Biotech Co., Ltd. (Shanxi, China); it had a molecular weight (MW) of 688.8 Da, a pH range of 4.0–6.0 (1 mg/mL in H_2_O), and a predicted density of 1.15 ± 0.06 g/cm^3^. Bovine serum albumin (BSA) was obtained from Solarbio Science & Technology Co., Ltd. (Beijing, China). Analytical-grade chemicals, including sodium hydroxide (NaOH), hydrogen peroxide (H_2_O_2_), acetic acid (CH_3_COOH), sodium acetate (CH_3_COONa), and calcium carbonate (CaCO_3_), were purchased from Macklin Biochemical Technology Co., Ltd. (Shanghai, China).

### 2.2. Microwave–Ultrasonic Cascade Pretreatment

#### 2.2.1. Microwave-Assisted Sodium Hydroxide Pretreatment

Dried reed straw powder (2.0 g) was accurately weighed into a reaction vessel. A NaOH solution was added at a solid-to-liquid ratio of 1: 20 (*w*/*v*), followed by thorough mixing. The mixture was subjected to microwave pretreatment in a microwave reactor. The effects of three variables were systematically investigated: NaOH concentration (0.5, 1.0, 1.5, 2.0, and 2.5% *w*/*v*), microwave power (100, 300, 500, and 700 W), and treatment time (5, 10, 15, 20, and 25 min). The reaction temperature was monitored in real time using a fiber-optic thermocouple and maintained at 80 °C via intermittent power adjustment; under the optimized conditions (500 W, 15 min), the temperature was stabilized at 80 ± 2 °C. The system operated under ambient pressure, and the power output was adjusted as needed to sustain the target temperature throughout each run. After pretreatment, solid residues were collected through a G3 sintered glass funnel. The residues were repeatedly rinsed with tap water until the filtrate became colorless and pH-neutral, then dried at 60 °C to constant weight for subsequent use.

#### 2.2.2. Ultrasonic-Assisted Hydrogen Peroxide Pretreatment

Samples pretreated with 1.0%, 1.5%, and 2.0% (*w*/*v*) NaOH (Section 2.2.1) were accurately weighed (1.0 g each) into reaction vessels. A NaOH solution was added at a solid-to-liquid ratio of 1: 20 (*w*/*v*) with thorough mixing. The mixtures underwent ultrasonic pretreatment in an ultrasonic reactor. The effects of three variables were systematically examined: H_2_O_2_ concentration (2, 5, and 8% *v*/*v*), ultrasonic power (100, 200, 300, 400, 500, and 600 W), and treatment time (5, 10, 15, 20, and 25 min). Solid residues were collected via a G3 sintered glass funnel, rinsed with tap water until the filtrate was colorless and pH-neutral, and dried at 60 °C to constant weight.

### 2.3. Enzymatic Hydrolysis

To analyze the effect of microwave–ultrasound cascade pretreatment, pretreated reed straw was mixed thoroughly with 0.05 M acetate buffer (pH 5.0) to achieve a 2% (*w*/*v*) solid–liquid ratio. Enzymatic hydrolysis was then performed in a rotary shaker at 50 °C and 180 rpm, using a cellulase loading of 20 filter paper units per gram of dry matter (FPU/g DM). For the construction of the enzyme cocktail system, enzymatic hydrolysis was conducted at an elevated solid–liquid ratio of 8% (*w*/*v*). The individual effects of varying additive levels on hydrolysis efficiency were systematically investigated: cellulase dosage (4, 8, 12, 16, and 20 FPU/g DM), β-glucosidase dosage (3, 6, 9, 12, and 15 FBG/g DM), sophorolipid dosage (20, 40, 60, 80, and 100 mg/g DM), and bovine serum albumin (BSA) dosage (20, 40, 60, 80, and 100 mg/g DM). Subsequently, the interactive effects of β-glucosidase, sophorolipid, and BSA on substrate hydrolysis were evaluated using an L_9_ (3^3^) orthogonal array design.

### 2.4. Characterization Analysis

The surface morphology of the reed straw samples, both before and after pretreatment, was characterized using a Sigma 300 scanning electron microscope (Carl Zeiss, Germany). Samples were uniformly coated onto conductive adhesive, subjected to three cycles of gold sputter coating, and observed at an accelerating voltage of 3.00 kV. Fourier transform infrared spectroscopy (FTIR) analysis was performed on a Nicolet iS20 spectrometer (Thermo Scientific, MA, USA) to identify characteristic functional groups and chemical bonds. For this, the sample and potassium bromide (KBr) were separately dried to a constant weight at 60 °C and 105 °C, respectively; mixed at an approximate mass ratio of 1:150; finely ground; pressed into pellets; and scanned over the wavenumber range 4000–400 cm^−1^ at a resolution of 2 cm^−1^ with 32 accumulations. Thermal stability was assessed using an SDT 650 thermogravimetric analyzer (TA Instruments, DE, USA). Samples were first dried at 80 °C for 2 h; then, approximately 5–10 mg was accurately weighed into an alumina crucible and heated under a nitrogen atmosphere (40 mL/min flow rate) from 100 °C to 550 °C at a rate of 10 °C/min while monitoring mass loss. Crystallinity was determined at room temperature using a D8 Advance 25 X-ray diffractometer (Bruker, Germany) by scanning over a 2θ range of 5° to 80° at 5°/min; the crystallinity index (CrI) was subsequently calculated according to Xu et al. [10]. Finally, specific surface area, pore size distribution, and pore volume were determined via Brunauer–Emmett–Teller (BET) analysis using an Autosorb iQ-2 instrument (Quantachrome Instruments, FL, USA), where approximately 0.3–0.4 g of sample was loaded into a clean, dry sample tube; degassed at 105 °C for 10 h; and subjected to adsorption measurements.

### 2.5. Fed-Batch Strategies for High-Solids Enzymatic Hydrolysis

Given that cellulase catalytic activity diminishes over prolonged reaction times, shorter substrate feeding intervals are beneficial for enhancing enzymatic hydrolysis efficiency. Therefore, all enzymes and additives (collectively equivalent to 25% *w*/*v* of the final solid loading) were added to the hydrolysis system at the outset. Under these conditions, initial solid loadings of 5–10% (*w*/*v*) were liquefied within 4–8 h. Consequently, fed-batch experiments employed an initial solid loading of 5–10% (*w*/*v*). Subsequently, after hydrolysis initiation for 5 h, the substrate was supplemented in 3–5 increments at 5 h intervals. Each supplement added 2–8% (*w*/*v*) substrate, continuing until the total solid loading reached 25% (*w*/*v*), with the total feeding duration not exceeding 20 h.

### 2.6. Sample Analysis

Chemical composition analysis of the reed straw samples was conducted according to the standard procedure established by the National Renewable Energy Laboratory [11]. In brief, the two-step acid hydrolysis procedure was as follows: samples (0.15 g) were treated with 1.5 mL of 72% (*w*/*w*) H_2_SO_4_ at 30 °C for 1 h under agitation (160 rpm), then diluted to 4% H_2_SO_4_ and autoclaved at 121 °C for 1 h. After neutralization with CaCO_3_ and filtration, the hydrolysate was subjected to HPLC analysis as described below. Acid-insoluble lignin was determined gravimetrically after incineration at 500 °C for 3 h. Prior to sugar quantification, samples were centrifuged at 12,000× *g* for 10 min, and the resulting supernatant was filtered through a 0.22 μm membrane filter (Jinteng Experimental Equipment Co., Tianjin, China). Sugar content was determined using high-performance liquid chromatography equipped with a Shodex sugar SH-1011 column (Showa Denko K.K., Tokyo, Japan) and a refractive index detector, employing a 50 mM aqueous sulfuric acid solution as the mobile phase at a flow rate of 0.5 mL/min. Glucose and xylose concentrations were quantified using external standard calibration curves. Analytical-grade D-glucose and D-xylose standards (Sigma-Aldrich, USA) were prepared at concentrations of 0.1, 0.25, 0.5, 1.0, 2.5, 5.0, and 10.0 g/L. Each standard was analyzed in triplicate, and the calibration curves were constructed by linear regression of peak area versus concentration. The resulting calibration equations were Area = 2.02 × 10^5^ × C(glucose) − 7.13 × 10^2^ (R^2^ = 0.9997) and Area = 2.05 × 10^5^ × C(xylose) − 1.71 × 10^3^ (R^2^ = 0.9994), where C is in g/L. For pretreated residues, the same analytical protocol was applied to solid fractions, while the liquid hydrolysates were directly filtered and analyzed by HPLC without acid hydrolysis. The time-scale fractal kinetic model was used to describe glucose release [12]. All data represent mean values ± standard deviations (SDs) derived from at least three independent replicate experiments, with statistical analysis performed using IBM SPSS Statistics software (version 19). Comparisons between two groups were analyzed using Student’s *t*-test, while multiple comparisons were analyzed by one-way analysis of variance (ANOVA) followed by Tukey’s honest significant difference (HSD) post hoc test, with a significance level set at *p* < 0.05. The fractal kinetic parameters (k and h) were estimated by non-linear regression using the Levenberg–Marquardt algorithm. The 95% confidence intervals for each fitted parameter were calculated based on the asymptotic standard errors. Goodness-of-fit was assessed by the coefficient of determination (R^2^) and adjusted R^2^.

(1) $$\mathrm{Glucan}\;\mathrm{recovery}\;\mathrm{rate}\;(\%)=\frac{M_{11}}{M_{01}}\times 100$$

(2) $$\mathrm{Xylan}\;\mathrm{removal}\;\mathrm{rate}\;(\%)=\frac{M_{02}-M_{12}}{M_{02}}\times 100$$

(3) $$\mathrm{Lignin}\;\mathrm{removal}\;\mathrm{rate}\;(\%)=\frac{M_{03}-M_{13}}{M_{03}}\times 100$$

(4) $$\mathrm{Glucose}\;\mathrm{yield}\;(\%)=\frac{M_{14}\times 0.9}{M_{11}}\times 100$$

(5) $$\mathrm{Glucose}\;\mathrm{recovery}\;\mathrm{rate}\;(\%)=\frac{M_{14}\times 0.9}{M_{01}}\times 100$$

(6) $$\mathrm{Xylose}\;\mathrm{yield}\;(\%)=\frac{M_{15}\times 0.88}{M_{12}}\times 100$$

(7) $$\mathrm{Solid}\;\mathrm{recovery}\;\mathrm{rate}\;(\%)=\frac{S_{1}}{S_{0}}\times 100$$

(8) $$\mathrm{CrI}\;(\%)=\frac{I_{002}-I_{\mathrm{am}}}{I_{002}}\times 100$$

(9) $$[G]=\frac{C_{G}[S_{0}]}{0.9}\times [1-\exp\;(\frac{-0.9\;k\;t^{1-h}}{1-h})]$$

M_01_–M_03_ and M_11_–M_13_ denote the masses of glucan, xylan, and lignin in the raw materials and pretreated residues, respectively. M_14_–M_15_ correspond to glucose and xylose masses in the enzymatic hydrolysate, with water loss coefficients of 0.9 (glucose-to-glucan) and 0.88 (xylose-to-xylan) applied to account for polymerization-induced mass changes. Substrate masses before and after pretreatment are designated S_0_ and S_1_. I_002_ and I_am_ denote the diffraction intensities corresponding to the crystalline region (2θ ≈ 22.5°) and amorphous region (2θ ≈ 18.0°), respectively. [G] is the glucose concentration at time t (h), C_G_ is the cellulose content in the substrate, k is the apparent rate constant, and h is the fractal index quantifying time-dependent hindrance from surface heterogeneity or diffusion limitations.

## 3. Results and Discussion

### 3.1. Microwave-Assisted Alkaline Pretreatment of Reed Straw

**Figure 1:**
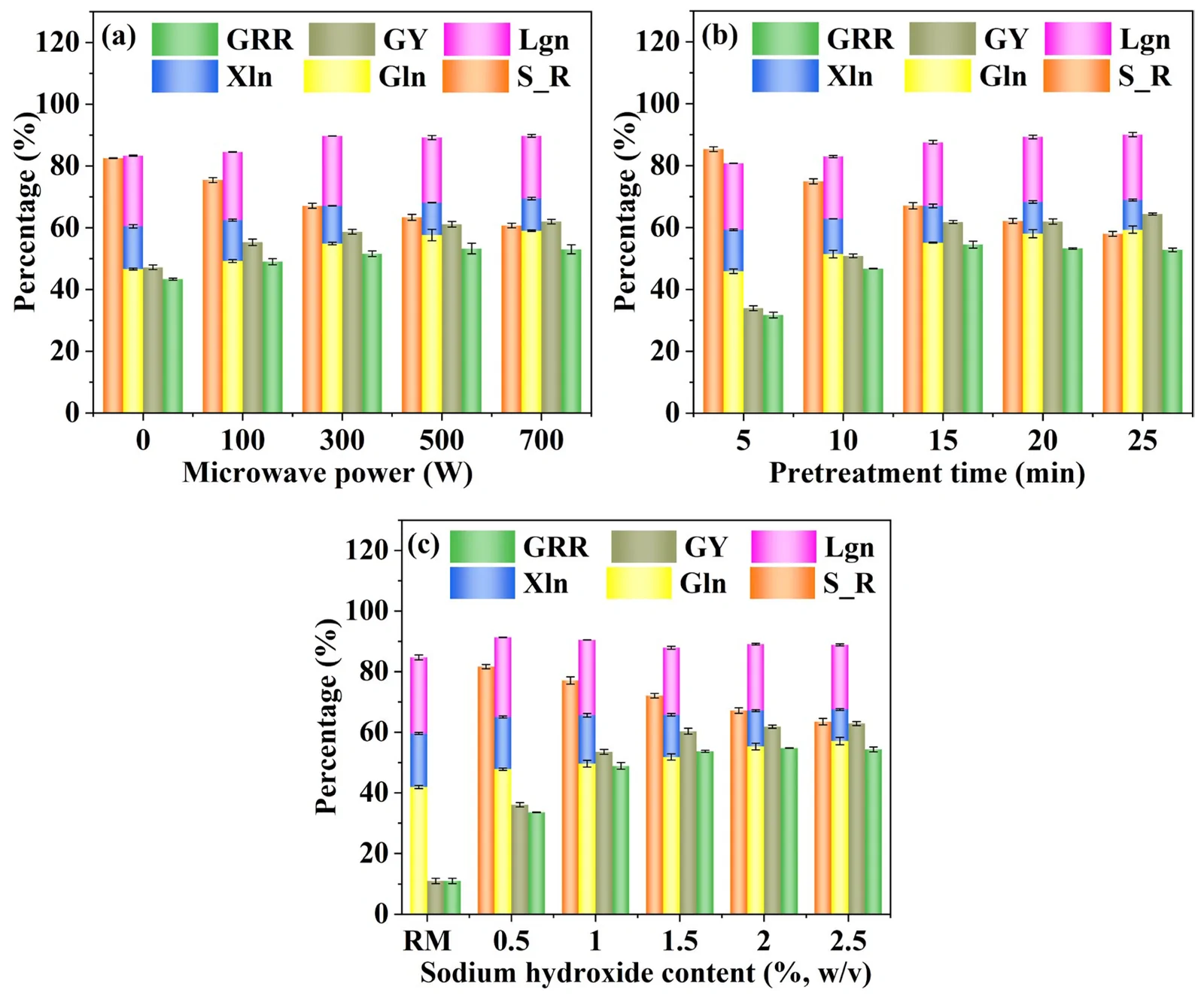
Effects of (**a**) microwave power, (**b**) pretreatment duration, and (**c**) NaOH concentration on chemical components and enzymatic hydrolysis of reed straw.

The systematic optimization of microwave power and duration was essential to maximize the efficiency of the alkali-assisted pretreatment. Increasing the microwave power from 0 W to 700 W significantly enhanced the removal of hemicellulose (from 35.29% to 64.20%) and lignin (from 24.87% to 50.87%), consequently reducing solid recovery from 82.54% to 60.72% (Figure 1a). This enhancement is attributed to the dielectric heating effect, where high-frequency oscillation of polar molecules accelerates the cleavage of ester/ether linkages within the lignin–carbohydrate complex (LCC) [13]. However, beyond an optimal threshold of 500 W, the marginal efficiency for component removal diminished sharply, and excessive energy began to degrade cellulose, reducing glucan recovery from 91.82% to 85.48%. Similarly, extending the pretreatment duration from 5 to 25 min progressively decreased solid recovery and increased component removal, with the substrate digestibility (glucose yield) peaking at 61.77% after 15 min (Figure 1b). Further extension offered negligible benefits while risking cellulose degradation and energy waste. The optimized conditions of 500 W for 15 min achieved a combined balance, maximizing glucose recovery (53.26%) by selectively removing lignin and hemicellulose while preserving cellulose integrity. This efficiency substantially surpasses conventional heating methods, as microwave-induced dielectric polarization and intense ion migration profoundly intensify reaction kinetics, rapidly cleaving key lignin bonds and hydrolyzing hemicellulose acetyl groups [14,15].

Concurrently, the NaOH concentration was optimized to balance chemical efficacy with minimal input and inhibitor formation. Increasing the alkali concentration from 0.5% to 2.5% (*w*/*v*) promoted the hydrolysis of LCC linkages and lignin sulfonation, leading to increased removal of hemicellulose (from 20.47% to 62.54%) and lignin (from 14.57% to 46.03%) and a corresponding rise in enzymatic glucose yield (Figure 1c). This aligns with the known mechanism where alkali disrupts the compact structure by saponifying cross-linking esters. However, excessive alkalinity (>1.5% NaOH) induced non-selective degradation, causing a decline in glucan recovery from 93.10% to 86.41% and elevating the risks of generating fermentation inhibitors and environmental burdens from waste streams. The optimal concentration of 1.5% NaOH achieved a peak glucose recovery of 53.72%, demonstrating that effective delignification can be accomplished under moderate alkali conditions when synergized with microwave irradiation. This integrated approach mitigates a key limitation of conventional alkaline pretreatment, which often requires high chemical loads, by leveraging microwave energy to enhance reagent penetration and reaction homogeneity, thereby shortening processing time and limiting chemical consumption.

### 3.2. Ultrasound-Assisted Hydrogen Peroxide Enhancement of Reed Straw Pretreatment

#### 3.2.1. Effect of Hydrogen Peroxide Concentration on Pretreatment Efficiency

Dilute alkali pretreatment removed some lignin and hemicellulose, but residual lignin–carbohydrate complexes still impeded enzymatic hydrolysis [16]. The effects of ultrasound-assisted H_2_O_2_ enhancement at different concentrations on the chemical composition and enzymatic digestibility of various alkali-pretreated residues were investigated (presented in Table 1). It is important to note that ultrasound treatment alone (without H_2_O_2_) on the alkali-pretreated residue resulted in negligible additional delignification (<3% lignin removal) and limited improvement in glucose yield, confirming that H_2_O_2_ is the essential oxidative agent and that ultrasound serves as a physical facilitator of ·OH generation and mass transfer. The following experiments therefore focused on the combined ultrasound–H_2_O_2_ treatment. For different alkali pretreated residues, increasing the H_2_O_2_ concentration from 2% to 8% (*v*/*v*) decreased the solid recovery rate by 5.42–7.39%. The removal rates for lignin, hemicellulose, and cellulose increased by 10.60–19.90%, 13.74–17.38%, and 2.50–3.45%, respectively. As the removal of lignin and hemicellulose enhances cellulose accessibility to cellulase, applying ultrasound-assisted H_2_O_2_ pretreatment increased substrate glucose yield by 8.45–9.46%. The optimal glucose recovery rate of 63.70 ± 0.63% was attained with 1.5% (*w*/*v*) microwave-assisted NaOH pretreatment followed by ultrasound-assisted 5% (*v*/*v*) H_2_O_2_ enhancement, achieving a corresponding glucose yield of 74.83 ± 1.34%. This represented an increase of 9.98% and 14.53% compared to alkali pretreatment only. During ultrasound-assisted H_2_O_2_ pretreatment, ultrasonic cavitation physically disrupts the ultrastructure of the alkali-pretreated lignocellulosic polymer complex, while simultaneously inducing efficient decomposition of H_2_O_2_ to generate highly reactive hydroxyl radicals (·OH). These radicals vigorously oxidize and degrade residual recalcitrant lignin (particularly cleaving β-O-4 ether linkages, the dominant inter-unit linkage in the lignin polymer, accounting for 45–60% of all linkages in grass lignins) and disrupt LCCs [6,17]. The selectivity of this process arises from the differential susceptibility of polymer components to ·OH attack: lignin, with its abundance of oxidizable aromatic rings and ether bonds, is preferentially degraded, and hemicellulose, with its amorphous structure and branched architecture, is also susceptible, whereas cellulose, with its densely packed crystalline domains stabilized by inter-chain hydrogen bonding, exhibits substantially higher resistance. This differential degradation underpins the measured enrichment of the cellulose fraction in the pretreated residue.

#### 3.2.2. Effect of Ultrasonic Power on Pretreatment Efficiency

**Figure 2:**
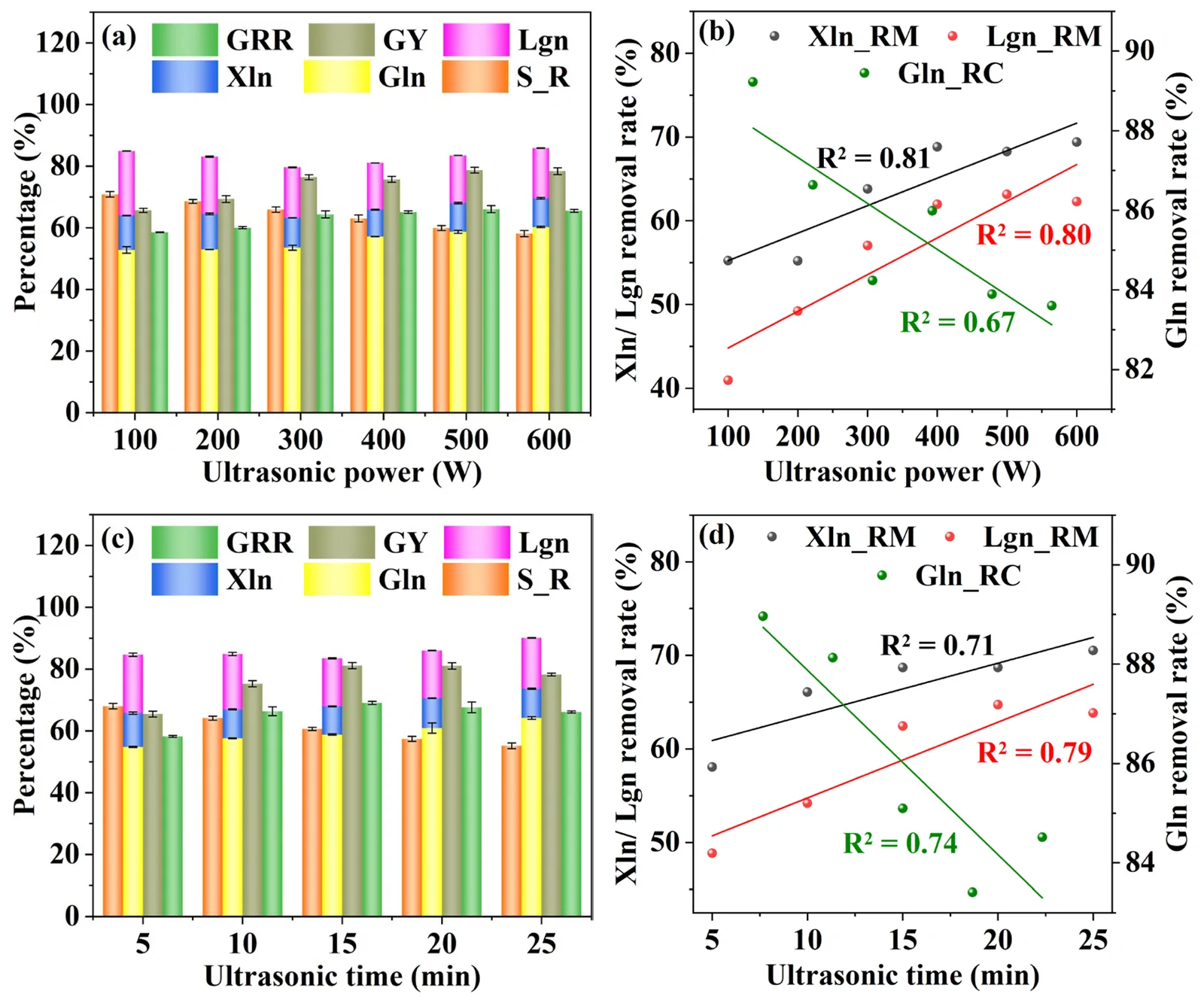
Effects of (**a**,**b**) ultrasonic power and (**c**,**d**) ultrasonic time on reed straw pretreatment efficiency and subsequent enzymatic saccharification performance.

The impact of ultrasonic power (100–600 W) on hydrogen peroxide-enhanced pretreatment of reed residue was investigated (Figure 2a). Lignin and hemicellulose further degraded (contents decreased from 20.94 ± 0.05% to 16.31 ± 0.07% and from 11.17 ± 0.09% to 9.32 ± 0.27%, respectively) as the ultrasonic power increased, while cellulose content increased from 52.79 ± 1.08% to 60.27 ± 0.24%. The removal of lignin and hemicellulose improved substrate accessibility to cellulase, and the glucose yield was increased from 65.65 ± 0.64% to 78.38 ± 1.11%. However, slight cellulose degradation also occurred, causing the glucose recovery rate to first increase and then decrease with rising power, peaking at 66.03 ± 1.14% at 500 W. Analysis (Figure 2b) revealed a weaker correlation between cellulose retention rate and ultrasonic power compared to the correlations for lignin and hemicellulose removal rates. This could be attributed to the enhancement of ultrasonic power intensifying cavitation, which (1) promotes interfacial delamination of LCCs and (2) increases hydroxyl radical (·OH) yield via H_2_O_2_ homolysis [4,18]. Lignin (rich in oxidizable groups) and amorphous hemicellulose are highly susceptible to ·OH, leading to rapid oxidative cleavage of bonds. In contrast, the dense crystalline structure of cellulose and its lack of readily oxidizable groups make it less vulnerable; radical attack primarily occurs in amorphous regions, resulting in substantially lower degradation compared to lignin and hemicellulose [19].

Ultrasonication duration significantly impacted the degradability of reed straw (illustrated in Figure 2c). As the duration increased from 5 min to 25 min, substantial removal of hemicellulose and lignin occurred (contents decreased from 10.91 ± 0.36% to 9.44 ± 0.17% and from 18.91 ± 0.55% to 16.46 ± 0.12%, respectively), while cellulose removal was less pronounced, resulting in an increase in its content from 54.83 ± 0.18% to 64.19 ± 0.43%. The removal rates of all three components were positively correlated with ultrasonication time, but the increments differed: lignin (15.00%) > hemicellulose (12.47%) > cellulose (4.45%) (Figure 2d). Prolonged ultrasonication enhanced cavitation effects and sustained ·OH generation, preferentially attacking and degrading structurally weaker regions like amorphous zones and chemical bonds with lower bond energy. Specifically, persistent cavitation microjets physically disrupted the LCC, while accumulated ·OH efficiently oxidized and degraded lignin (particularly cleaving β-O-4 linkages) and the amorphous regions of hemicellulose [20]. Conversely, cellulose was resistant to degradation due to the stability of its crystalline regions and the inertness of its side-chain groups towards ·OH. During the initial pretreatment phase, readily degradable components (lignin side chains and hemicellulose branches) reacted rapidly. Later, the increased recalcitrance of remaining structures (condensed lignin and crystalline regions) led to a decline in the increment of removal rates for all components as ultrasonication duration extended [11]. Although increased hemicellulose and lignin removal favored subsequent enzymatic hydrolysis, cellulose degradation during this process also caused glucose loss. Consequently, the highest glucose recovery rate (69.03 ± 0.54%) after enzymatic hydrolysis was achieved at an optimal ultrasonication duration of 15 min. This represented a 1.28-fold and 6.29-fold increase compared to microwave-assisted alkaline pretreated residue and raw material, respectively.

### 3.3. Structural Characterization

#### 3.3.1. Surface Morphology

**Figure 3:**
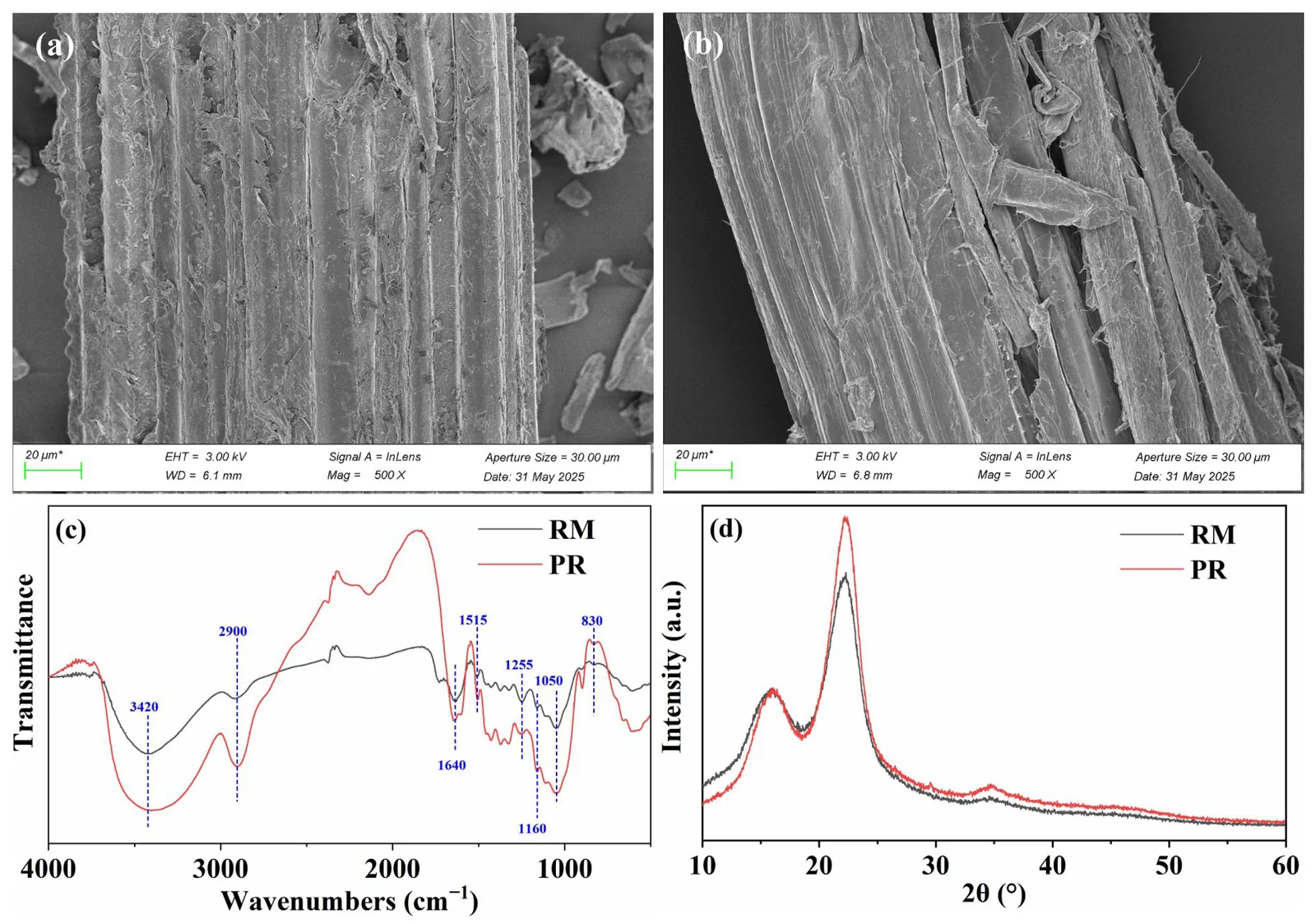
Comparative analysis of surface morphology, functional groups, and crystallinity in raw and pretreated biomass residues via SEM ((**a**) raw material; (**b**) pretreated residue), FT-IR (**c**), and XRD (**d**).

Scanning electron microscopy (SEM) analysis demonstrates that reed straw raw material exhibits a dense, smooth surface with fiber bundles tightly enveloped by an amorphous matrix (Figure 3a). Following stepwise pretreatment (microwave-assisted alkali + ultrasound-assisted H_2_O_2_), extensive delamination of the amorphous surface layer exposes parallel-aligned fiber bundles (Figure 3b), while deeply eroded groove-like pores (5–10 μm in diameter) form on fiber surfaces. Reduced inter-fiber adhesion and partial microfiber dissociation confirm degradation-resistant barrier disruption. These structural transformations directly correspond to compositional changes—substantial removal of lignin/hemicellulose drives matrix clearance and pore formation, whereas high cellulose retention preserves fiber skeleton integrity without structural fracturing. Collectively, SEM evidence verifies that this pretreatment strategy enhances fiber accessibility through targeted delignification and disruption of the dense architecture, providing direct morphological validation for compositional analyses and enzymatic efficiency improvement.

#### 3.3.2. Functional Groups, Crystalline Properties and Pore Structure of Different Samples

Functional group characterization was performed using Fourier transform infrared (FTIR) spectroscopy. The observed absorption bands and their corresponding assignments are detailed as follows (presented in Figure 3c): the broad band at 3420 cm^−1^ is attributed to O-H stretching vibrations (primarily from the cellulose glucose ring); the band at 2900 cm^−1^ corresponds to C-H stretching vibrations (from methyl and methylene groups); bands at 1640 cm^−1^ and 1515 cm^−1^ are characteristic of aromatic skeleton vibrations (associated with lignin). Additional notable peaks include: C-O/C=O vibrations at 1255 cm^−1^, C-O-C pyran ring stretching and/or aliphatic C=O stretching at 1160 cm^−1^, C-O deformation vibrations (from secondary alcohols and aliphatic ethers) at 1050 cm^−1^, and syringyl C-H vibrations at 830 cm^−1^. Comparative analysis of transmittance spectra revealed significant changes in the pretreated samples. Specifically, a reduction in lignin content and alterations in lignin subunit composition were indicated by changes in the characteristic lignin bands (1640 cm^−1^ and 1515 cm^−1^) for samples subjected to the cascaded microwave–alkaline/ultrasonic–H_2_O_2_ pretreatment. Concurrently, evidence for cellulose enrichment was provided by the decreased transmittance observed at bands characteristic of cellulose, particularly the O-H stretching region (3420 cm^−1^) [19,21].

XRD analysis demonstrates that cascaded microwave–alkaline and ultrasonic–H_2_O_2_ pretreatment apparently increased biomass crystallinity index (CrI) from 58.77% to 69.16% and cellulose content from 42.90% to 58.80%, while reducing the CrI/cellulose ratio from 1.37 to 1.18 (Figure 3d and Table 2). This shift primarily results from selective removal of non-cellulosic components (lignin and hemicellulose), exposing crystalline cellulose regions. Critically, the 13.9% decrease in CrI/cellulose ratio confirms that the apparent CrI elevation is attributed to the removal of non-cellulosic components rather than to enhanced cellulose crystallinity. The process aligns with the pretreatment mechanism: microwave–alkali disrupts lignin–carbohydrate linkages and hydrolyzes hemicellulose, while ultrasound–H_2_O_2_ generates ·OH via cavitation to cleave lignin β-O-4 bonds, collectively dismantling amorphous barriers to enhance apparent cellulose crystallinity [22,23]. Triangulated evidence from glucose yield, XRD, and compositional analyses verifies synergistic optimization of lignocellulosic conversion efficiency through targeted recalcitrance deconstruction.

**Figure 4:**
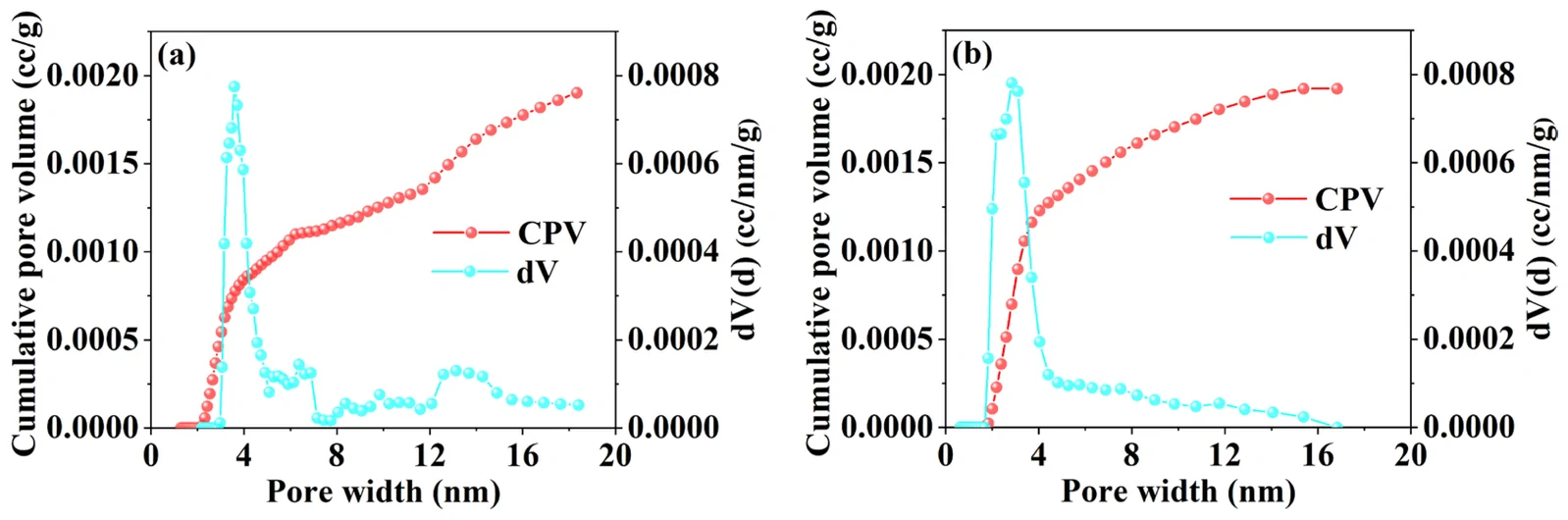
BET analysis the pore structure of reed straw: (**a**) raw material; (**b**) pretreated residue.

Figure 4 and Table 2 demonstrate the impact of pretreatment on reed straw pore structure, where synergistic mechanisms—microwave-induced internal “explosion”, alkali swelling disrupting lignin–carbohydrate complexes, H_2_O_2_ oxidation, and ultrasonic cavitation—significantly restructured the pore system of the substrate [24,25]. The specific surface area increased by 32% (from 0.81 m^2^/g to 1.07 m^2^/g), indicating effective removal of the lignin–hemicellulose barriers encasing cellulose microfibrils, thereby exposing previously buried cellulose surfaces and increasing accessible catalytic sites for cellulases. The average pore diameter showed only a slight increase (from 2.77 nm to 2.84 nm). It should be noted that these pore dimensions are considerably smaller than typical cellulase sizes (approximately 4.4–6.6 nm in diameter); therefore, the improved hydrolysis is more plausibly attributed to the increased external surface area available for enzyme adsorption rather than to enhanced enzyme penetration into micropores [26]. Nevertheless, the increased surface area, together with the removal of pore-blocking debris, may facilitate enzyme access to the substrate surface and partially alleviate diffusional constraints. Alkali swelling widened existing channels and cleared micro-debris (hemicellulose degradation products) from pores, further enhancing enzyme mass transfer into the polymer matrix. The invariance of total pore volume (0.02 cm^3^/g) confirms that the pretreatment primarily activates previously closed mesopores (2–50 nm) rather than creating new macropores, consistent with the interpretation that the polymer composite undergoes structural reorganization rather than bulk disintegration. This pore restructuring thus simultaneously overcomes two key physical limitations in enzymatic hydrolysis: the increased surface area boosts enzyme binding site density, while the slight pore widening combined with pore clearance synergistically lowers enzyme diffusion barriers, collectively establishing an efficient enzyme–substrate interface that provides the structural foundation for significantly enhanced hydrolysis efficiency [27].

### 3.4. Impact of Cellulolytic Enzymes and Non-Enzymatic Additives on Substrate Saccharification Efficiency

**Figure 5:**
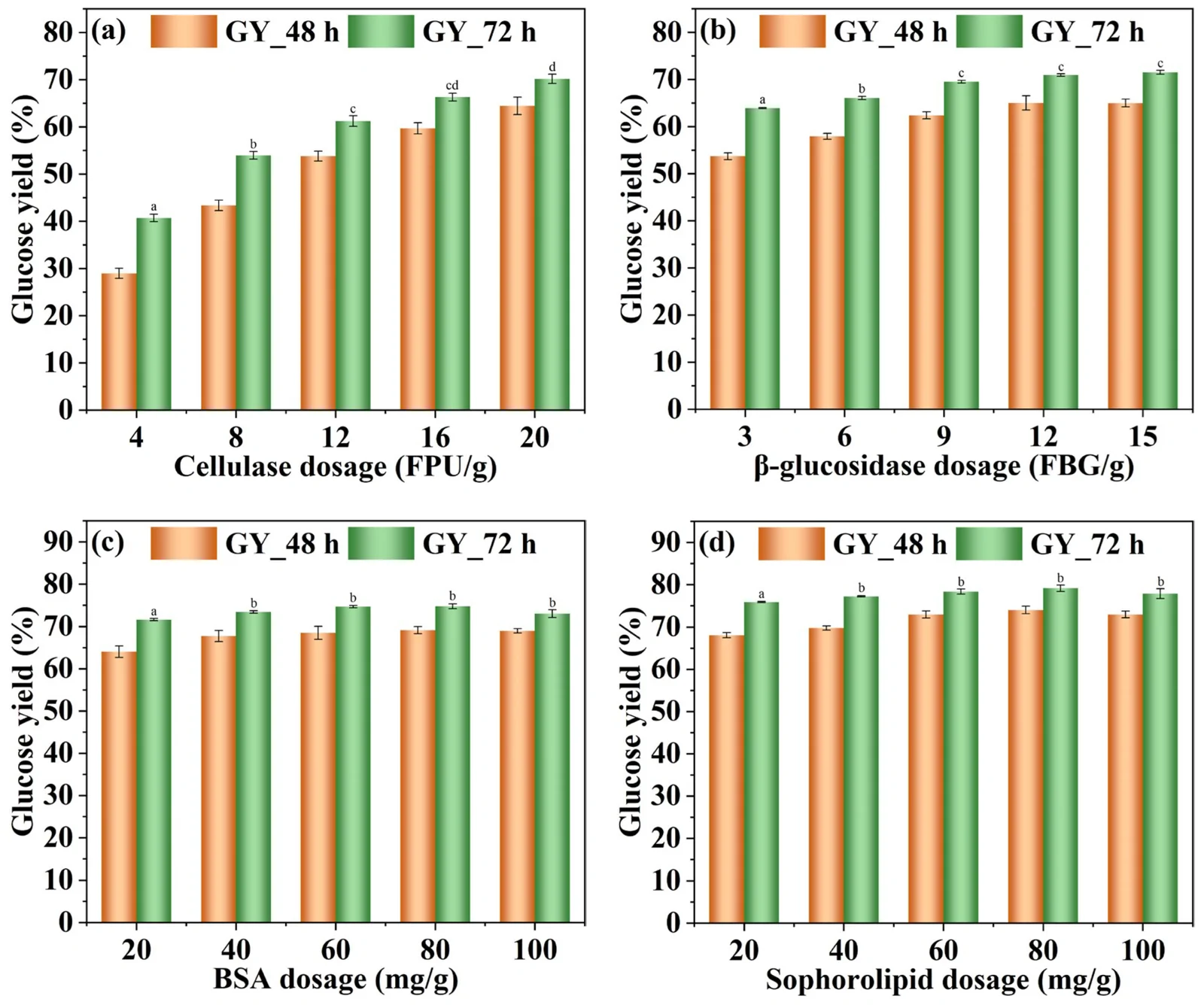
The dose–response effects of (**a**) cellulase loading and key additives ((**b**) β-glucosidase, (**c**) BSA, and (**d**) sophorolipid) on enzymatic glucose yield from reed residue. Different letters above the bars indicate statistically significant differences (*p* < 0.05); bars sharing the same letter (including composite letters such as ab) are not significantly different (*p* ≥ 0.05).

To overcome the core bottleneck where enzyme costs constitute 20–40% of total biomass conversion expenses, implementing low cellulase-loading saccharification via combined enzyme-additive systems is imperative. As shown in Figure 5a, increasing cellulase loading from 4 to 20 FPU/g notably enhanced glucose yield from 40.73 ± 0.79% to 70.19 ± 0.99%, due to improved coverage of cellulose active sites accelerating initial hydrolysis. Increasing cellulase loading to 12 FPU/g achieved a yield of 61.24% (a 20.51% increase over 4 FPU/g); however, further enzyme addition, while still enhancing yield, saw the growth rate drop from 13.24% to 3.87%. This indicates that enzyme excess led to higher free enzyme proportion and diminishing returns in enzyme–substrate binding efficiency, revealing that this loading essentially meets the saturation adsorption requirement on cellulose surfaces. Consequently, 12 FPU/g was selected as the baseline loading. A complementary degradation system was then established by incorporating β-glucosidase to alleviate product inhibition and non-enzymatic additives to minimize non-productive adsorption, significantly reducing enzyme usage while maintaining efficiency.

The necessity of β-glucosidase stems from its role in mitigating cellobiose-induced inhibition of exoglucanases, a key kinetic bottleneck. Exogenous β-glucosidase converts cellobiose to glucose, eliminating inhibition while promoting continuous cellulase action through localized product concentration reduction [28]. As shown in Figure 5b, increasing β-glucosidase loading from 3 to 9 FBG/g raised the glucose yield by 5.61% (from 63.94 ± 0.69% to 69.54 ± 0.86%) by relieving competitive inhibition. Beyond 9 FBG/g, yield increments sharply declined (1.40% at 12 FBG/g; 0.58% at 15 FBG/g) due to a reaction kinetics plateau from depleted cellobiose and reduced enzyme–substrate contact. The optimal dose of 9 FBG/g was determined based on (1) a marked inflection point that occurred where the yield growth rate declined by 59.41% when enzyme loading increased from 6 to 9 FPU/g, compared to the increment from 9 to 12 FPU/g, and (2) maximized productivity per enzyme unit (1.15%/FBG increment), representing the most cost-effective dosage beyond which marginal gains diminished notably.

Figure 5c shows a non-linear dose–response relationship between BSA loading and glucose yield. Increasing BSA from 20 mg/g to 60 mg/g enhanced glucose yield by 3.07% at 72 h and 4.46% at 48 h, indicating accelerated substrate hydrolysis. However, further BSA increases yielded diminishing returns or slight yield reductions. BSA exhibits a dual effect: at optimal levels (≤60 mg/g), it reduces non-specific cellulase adsorption onto lignin via steric hindrance, maintains enzyme conformational stability, and promotes productive enzyme–substrate binding. Conversely, excess BSA (>80 mg/g) competes with endo/exoglucanases for cellulose binding sites and increases solution viscosity, impeding enzyme–substrate diffusion (especially in high-solids hydrolysis), thereby worsening hydrolysis kinetics [29,30]. Consequently, 60 mg/g is identified as the critical threshold, achieving a hydrolysis rate of 74.70 ± 0.46%.

The impact of SL loading on glucose yield is shown in Figure 5d. Increasing SL from 20 mg/g to 80 mg/g raised yields by 3.24% at 48 h and 5.94% at 72 h, indicating its effectiveness in maintaining the stability of enzymatic catalytic activity. However, further increasing to 100 mg/g reduced the 72 h yield to 77.90%, establishing a biphasic dose–response curve. This is attributed to the amphiphilic nature of SL: at optimal levels (≤80 mg/g), it reduces liquid–solid interfacial tension, facilitating enzyme–substrate contact. The hydrophilic sugar moiety binds cellulases, while the hydrophobic chain inserts into the hydrophobic regions of the substrate, forming an “enzyme–SL–substrate” ternary complex [14]. This alleviates non-productive lignin adsorption and enhances catalytic activity, achieving a peak yield of 79.17%. Excess SL (>80 mg/g), however, forms micelles that entrap free enzymes (causing conformational inactivation) and clog substrate pores, reducing accessibility [7]. This explains the 1.27% yield decrease at 100 mg/g compared to the peak.

### 3.5. Construction of a High-Efficiency Enzyme-Additive System

The ratios of β-glucosidase, sophorolipid, and bovine serum albumin were systematically optimized via orthogonal experiments to enhance the glucose yield from enzymatic saccharification of pretreated reed straw residue (shown in Table 3). The results demonstrated that using code 8 (BGL: 12 FBG/g, sophorolipid: 60 mg/g, BSA: 40 mg/g) achieved a glucose yield of 81.58 ± 0.73% at 72 h, representing a 20.34% increase over the control (no β-glucosidase and additives; *p* < 0.05). This performance substantially surpassed the enhancement effect of any single component, demonstrating the beneficial combination of these three additives. Orthogonal analysis revealed the contribution order as BGL > sophorolipid > BSA. The core role of BGL lies in efficiently relieving cellobiose inhibition on cellulases; sophorolipid enhances enzyme–substrate contact efficiency by reducing liquid–solid interfacial tension; and BSA inhibits the non-productive adsorption of enzymes onto lignin. Based on this contribution analysis, the theoretically optimal combination was adjusted to BGL: 12 FBG/g, sophorolipid: 60 mg/g, and BSA: 60 mg/g. Validation experiments confirmed a further increased glucose yield of 82.32 ± 0.85%. In conclusion, the combination of BGL, sophorolipid, and BSA exhibits a pronounced promoting effect on the saccharification of pretreated reed residue, and orthogonal optimization enables further enhancement of this combined effect.

### 3.6. Effects of Non-Enzymatic Additives on Enzymatic Hydrolysis Efficiency and Kinetic Characteristics

**Figure 6:**
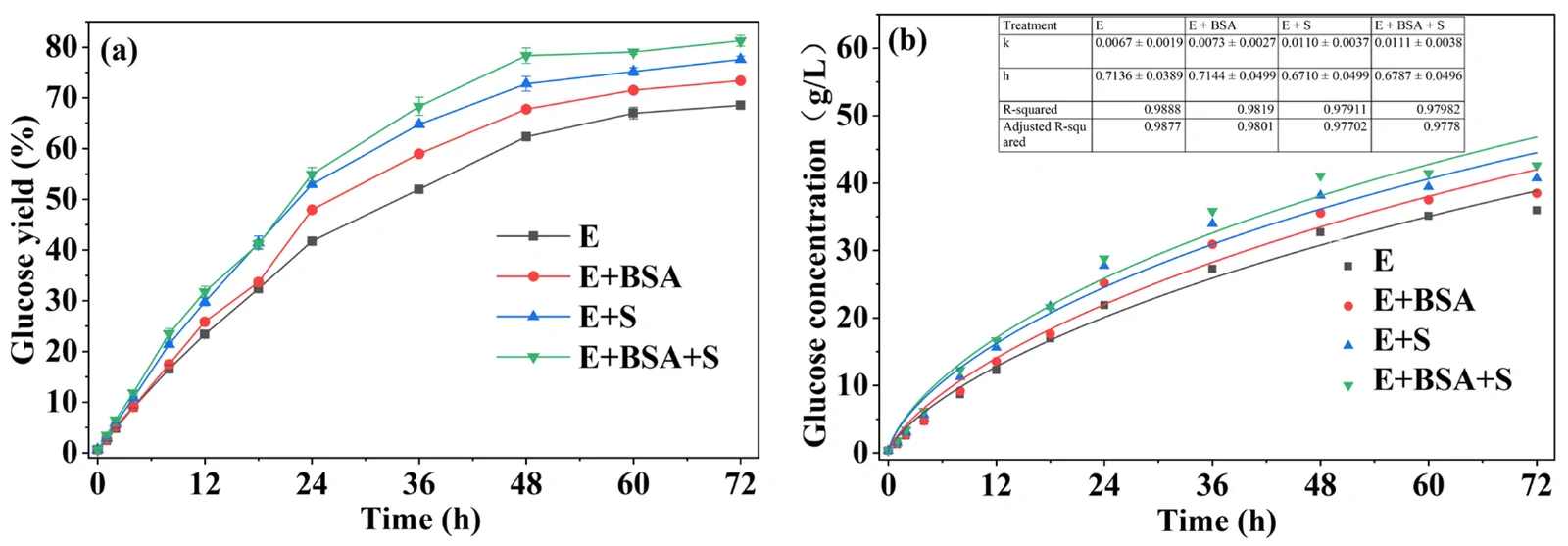
Time profiles of (**a**) glucose yield during enzymatic hydrolysis of pretreated reed straw with cellulase alone or supplemented with BSA, sophorolipid, or their combination. (**b**) Fractal-like kinetic parameters (rate constant “k” and fractal dimension “h”) derived from fitting the hydrolysis data.

The effects of bovine serum albumin (BSA) and sophorolipid, alone and in combination, as non-enzymatic additives on the saccharification efficiency of lignocellulosic substrates were evaluated (Figure 6a). During enzymatic hydrolysis, glucose yields in all treatment groups increased with reaction time, albeit with a faster initial rate followed by a gradual deceleration. The order of enhancement was consistently cellulase + BSA + sophorolipid > cellulase + sophorolipid > cellulase + BSA > cellulase alone. In the early stage (8–24 h), the additives exhibited the most pronounced effects: at 8 h, the sophorolipid and combined groups improved yields by 29.3% and 41.7%, respectively, compared with the control (enzymes-only), whereas the BSA group showed only a 5.6% increase; by 24 h, the corresponding improvements were 26.8%, 31.6% and 14.8%. These results indicate that sophorolipid substantially accelerates saccharification during the initiation phase, while BSA plays a relatively limited role. In the later stage (48–72 h), the relative increments narrowed: at 72 h, the combined, sophorolipid and BSA groups exhibited increases of 18.5%, 13.2% and 7.1%, respectively, suggesting that the marginal benefits of the additives diminish as readily hydrolyzable substrates are depleted and product accumulation exacerbates feedback inhibition, ultimately leading to saturation of the enhancement. The observed promotion can be attributed to the mitigation of non-productive lignin–cellulase adsorption. As a biosurfactant, sophorolipid weakens hydrophobic and electrostatic interactions between lignin and enzymes, thereby reducing non-productive binding. BSA, on the other hand, preferentially occupies non-specific binding sites on the enzyme, preventing futile adsorption, while also stabilizing cellulase activity and alleviating product-induced inhibition [8,31].

To further elucidate the underlying mechanism, a fractal-like kinetic model was employed to fit the enzymatic hydrolysis profiles. This model effectively describes the complex kinetic behavior of heterogeneous reactions such as cellulase-catalyzed saccharification. As shown in Figure 6b, the rate constant, k, for the cellulase-only treatment was 0.0067 ± 0.0019; it increased slightly to 0.0073 ± 0.0027 upon BSA addition, rose markedly to 0.0110 ± 0.0037 with sophorolipid, and reached the highest value of 0.0111 ± 0.0038 for the combined treatment, which is fully consistent with the order of saccharification enhancement. Concurrently, the fractal dimension, h, decreased from 0.7136 ± 0.0389 in the control to 0.7144 ± 0.0499 (BSA), 0.6710 ± 0.0499 (sophorolipid), and 0.6787 ± 0.0496 (combined). The reduction in h signifies diminished surface structural heterogeneity of the substrate and alleviated diffusional constraints during hydrolysis (sophorolipid markedly lowered h), whereas BSA exerted a negligible effect on h but marginally increased k. All models exhibited excellent goodness-of-fit (R^2^ > 0.98, adjusted R^2^ > 0.97), confirming the reliability of the fitting. The kinetic parameters corroborate the saccharification data: sophorolipid enhances hydrolysis by both increasing the reaction rate and improving substrate accessibility, while BSA primarily serves as an auxiliary enzyme-stabilizing agent.

### 3.7. Impact of Different Fed-Batch Strategies on High-Solids Enzymatic Hydrolysis

High-solids enzymatic hydrolysis (>15% *w*/*v*) effectively generates sugar-rich hydrolysates but elevates system viscosity, impairing heat/mass transfer and hindering hydrolysis efficiency. Fed-batch strategies counteract this “solid effect” by incremental substrate addition, enhancing mixing efficiency [20]. Table 4 details fed-batch strategies and hydrolysis results for pretreated reed residue at 25% *w*/*v* solids loading, showing that increased feeding frequency within a given period (20 h) enhances substrate conversion efficiency. After 120 h of enzymatic digestion, mode 7 demonstrated the highest glucose yield (78.54 ± 1.04%), surpassing batch hydrolysis (mode 1) by a significant margin of 26.71% (*p* < 0.05). This protocol involves single addition of all enzymes/non-enzymatic factors initially, followed by sequential feeding of 8% *w*/*v* (0 h), 6% *w*/*v* (5 h), 4% *w*/*v* (10 h), 4% *w*/*v* (15 h), and 3% *w*/*v* (20 h) substrates, yielding 128.71 ± 1.71 g/L glucose. Its core merit lies in staged solids control: an initial low load (8% *w*/*v*) ensures efficient enzyme–substrate mixing and reaction initiation, while gradient feeding sustains low viscosity to prevent mass transfer barriers from localized substrate overload.

### 3.8. Mass Balance for Glucose Conversion from Reed Straw

**Figure 7:**
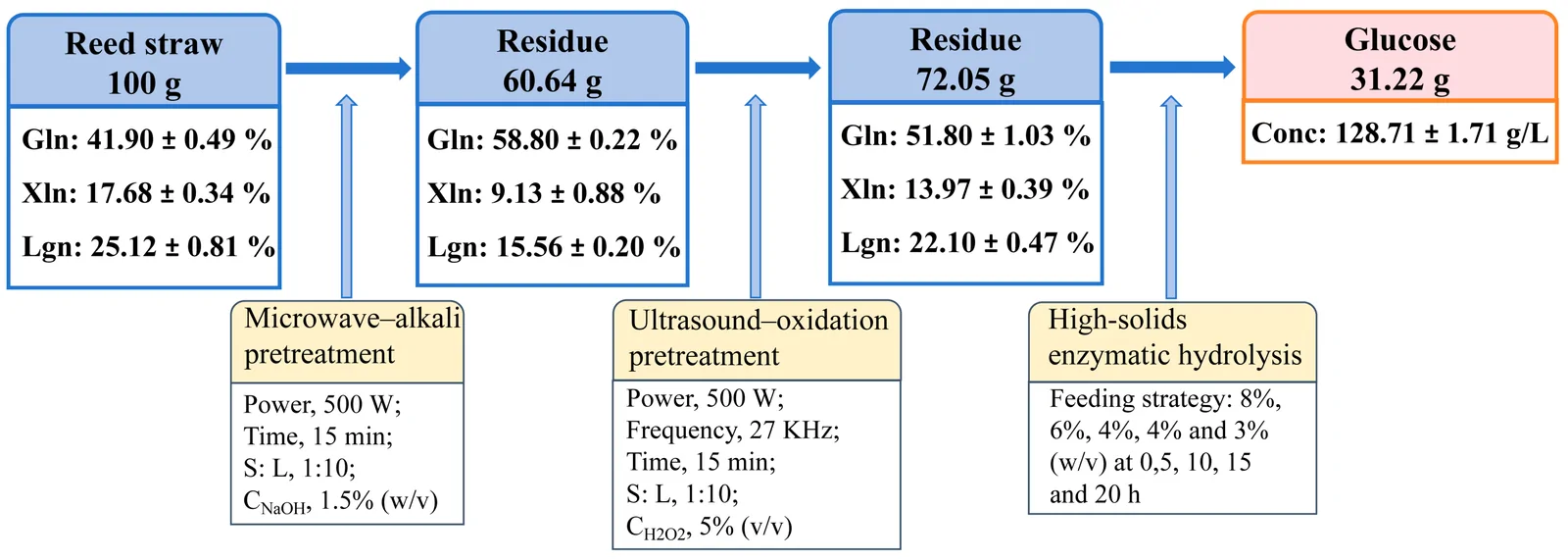
Mass balance analysis of the glucose production from reed straw.

This study establishes an integrated sugar-production platform for reed straw valorization, as summarized in Figure 7. Starting from 100 g of raw material, sequential process optimization enabled efficient fractionation and high-titer glucose production under mild conditions. First, combined microwave-assisted NaOH and ultrasound-assisted H_2_O_2_ pretreatment effectively disrupted lignocellulosic recalcitrance while minimizing alkali input, yielding 72.05 g of solids with enriched cellulose content and improved substrate accessibility. Subsequent enzymatic hydrolysis at 25% (*w*/*v*) solids using a tailored multi-enzyme cocktail achieved a conversion efficiency of 78.54 ± 1.04% after 120 h (based on the glucan content in the pretreated residue), despite a reduced cellulase dosage of only 12 FPU/g. This corresponds to a total glucose recovery of 31.22 g per 100 g of raw reed straw, calculated on the basis of the initial glucan content (approximately 41.9 g glucan per 100 g raw material), representing a glucan-to-glucose conversion efficiency of approximately 74.5% based on the original biomass.

### 3.9. Benchmarking with Previously Reported Pretreatment Strategies for Reed Straw

To contextualize the performance of the integrated cascade pretreatment and additive-boosted saccharification strategy, a comparison was made with recently reported pretreatment approaches for reed straw (Table 5). These include hydrothermal pretreatment (HTP) [32], citric acid pretreatment (CAP) [32], ethanol-assisted lactic acid pretreatment [33], deep eutectic solvent (DES) pretreatment [34], sodium sulfite–ethanolamine synergistic pretreatment [35], salicylic acid–ethylene glycol/water (SA-EG/H_2_O) pretreatment [36], and liquid hot water–ammonia/oxygen (LHW-NH_3_·H_2_O/O_2_) pretreatment [37].

Several observations emerge from this comparison. First, most of the reported strategies achieve high glucose yields (77.5–98.8%) but typically require elevated temperatures (120–180 °C) and prolonged treatment times (40–120 min), often under pressurized conditions [32,33,34,35]. In contrast, the microwave–alkali and ultrasound–H_2_O_2_ cascade described in this study operates under ambient pressure without hazardous steam explosion risks, thereby improving process safety and operating under milder conditions. Second, while several studies employ surfactant additives such as Tween 80 or PEG 6000 to boost saccharification, the use of sophorolipid—a biodegradable biosurfactant—combined with BSA offers a more environmentally benign alternative [32,34,37]. Notably, sophorolipid has been demonstrated to enhance the enzymatic hydrolysis efficiency of alkali-pretreated reed straw by 30.65%, and its synergy with tea saponin can further elevate efficiency to 99.56% [38]. The results obtained in this study confirm that the binary sophorolipid/BSA supplementation achieves a 20.34% improvement over the additive-free control at low cellulase loadings, validating the effectiveness of this biosurfactant-based strategy.

Third, many of the high-performance pretreatments listed in Table 5 rely on relatively high enzyme loadings (most are 20 FPU/g substrate) to attain their reported yields. In the present process, a glucose titer of 128.71 ± 1.71 g/L and a yield of 78.54 ± 1.04% were achieved at 25% (*w*/*v*) solids under fed-batch operation, corresponding to 31.22 g glucose per 100 g raw material—all at a reduced cellulase dosage of 12 FPU/g enabled by the combined additive formulation. While the absolute glucose yield is somewhat lower than the highest values reported in the literature, this trade-off is achieved under substantially milder conditions (ambient pressure: 80 °C) and with lower enzyme inputs, which is favorable for practical biorefinery economics [35,36]. Collectively, this benchmarking analysis suggests that the integrated strategy offers a pragmatic pathway for reed straw valorization, reconciling operational simplicity with promising saccharification performance. It should be noted, however, that this study was conducted at laboratory scale, and a comprehensive techno-economic assessment or life-cycle analysis was beyond its scope. Quantitative validation through process simulation and scale-up studies is warranted before drawing definitive conclusions regarding industrial feasibility or overall sustainability. Nonetheless, the experimental data presented herein provide a solid foundation for such future evaluations.

## 4. Conclusions

This study established an integrated system of cascade pretreatment, dual-additive saccharification, and fed-batch high-solids hydrolysis that achieves efficient conversion of reed straw into fermentable sugars. The microwave–ultrasound cascade pretreatment, operated under mild, pressure-free conditions, increased enzymatic efficiency by 1.28-fold relative to single-stage pretreatment, confirming the feasibility of physical-field coupling for enhancing performance while reducing severity. Fractal kinetic analysis confirmed that the combined action of BSA and sophorolipid increases the initial hydrolysis rate and alleviates diffusional constraints (increased k and reduced h). The finding that h was slightly lower with sophorolipid alone suggests that BSA contributed little to surface homogenization, supporting their complementary rather than synergistic roles. These kinetic insights provide a quantitative basis for additive design in low-enzyme-loading saccharification. At the engineering scale, fed-batch operation at 25% (*w*/*v*) solids pushed the glucose titer beyond the industrial threshold of 100 g/L (reaching 128.71 g/L), corresponding to a total glucose recovery of 31.22 g per 100 g raw material. This performance, attained with markedly reduced chemical and enzyme inputs, suggests the process’s potential for economic viability. Collectively, this work provides a viable route for reed straw valorization and establishes a multi-scale integrated framework for efficient, low-input conversion of other recalcitrant lignocellulosic feedstocks.

## Figures and Tables

**Table 1:** The influence of hydrogen peroxide concentration on the effect of ultrasound-assisted pretreatment.

C_NaOH_	C_H_2_O_2__	S_R (%)	Gln (%)	Gln_RC (%)	Xln (%)	Xln_RM (%)	Lgn (%)	Lgn_RM (%)	GY (%)	GRR (%)
1	0	75.81 ± 0.93	49.68 ± 0.53	89.87 ± 0.14	15.92 ± 0.64	31.79 ± 19.2	24.90 ± 0.38	24.87 ± 0.23	54.67 ± 0.73	49.14 ± 0.74
	2	73.01 ± 0.92	51.41 ± 0.77	89.40 ± 1.89	15.39 ± 0.07	38.22 ± 1.69	24.53 ± 0.64	29.15 ± 0.95	60.54 ± 0.89	54.14 ± 1.94
	5	69.80 ± 0.53	53.69 ± 1.00	89.25 ± 0.71	13.01 ± 0.53	44.75 ± 1.04	23.04 ± 0.15	36.34 ± 0.89	68.35 ± 1.00	61.01 ± 1.37
	8	67.59 ± 0.56	53.98 ± 0.66	86.90 ± 0.14	11.96 ± 0.53	55.60 ± 0.75	22.52 ± 0.40	39.75 ± 0.56	70.00 ± 0.69	60.82 ± 0.50
1.5	0	69.81 ± 0.83	52.61 ± 0.95	87.62 ± 0.54	14.13 ± 0.38	44.21 ± 0.84	22.14 ± 0.53	38.500.76	61.85 ± 0.62	54.20 ± 0.88
	2	67.72 ± 0.91	53.66 ± 0.92	86.55 ± 1.31	13.18 ± 0.38	50.93 ± 0.97	21.82 ± 0.03	41.53 ± 0.70	67.73 ± 0.71	58.62 ± 0.27
	5	63.61 ± 0.85	56.20 ± 0.77	85.14 ± 0.69	11.70 ± 0.53	59.13 ± 0.94	17.10 ± 0.50	56.97 ± 0.68	74.83 ± 1.34	63.70 ± 0.63
	8	60.40 ± 0.66	57.78 ± 1.53	83.10 ± 0.27	10.18 ± 0.23	66.20 ± 0.79	16.61 ± 0.54	60.32 ± 0.85	76.18 ± 0.88	63.31 ± 0.94
2	0	66.55 ± 0.85	54.14 ± 1.14	85.97 ± 0.72	11.24 ± 0.35	57.70 ± 0.78	21.55 ± 0.06	42.90 ± 0.88	63.97 ± 1.41	54.98 ± 0.75
	2	64.75 ± 0.87	54.17 ± 1.09	83.53 ± 1.07	10.52 ± 0.22	62.57 ± 1.04	20.78 ± 0.08	46.76 ± 0.52	70.28 ± 0.96	58.71 ± 1.51
	5	60.14 ± 0.71	57.26 ± 0.71	82.02 ± 0.43	9.23 ± 0.34	69.50 ± 0.39	16.64 ± 0.49	60.40 ± 0.69	74.66 ± 0.91	61.25 ± 1.07
	8	56.38 ± 0.73	59.80 ± 1.55	80.27 ± 0.48	7.64 ± 0.15	76.31 ± 0.69	14.94 ± 0.20	66.66 ± 0.87	77.98 ± 0.53	62.59 ± 0.06

Footnotes: C_NaOH_, NaOH concentration; C_H_2_O_2__, H_2_O_2_ concentration; S_R (%), solid recovery rate; Gln (%), glucan con-tent; Gln_RC (%), glucan recovery rate; Xln (%), xylan content; Xln_RM (%), xylan removal rate; Lgn (%), lignin content; Lgn_RM (%), lignin removal rate; GY (%), glucose yield; GRR (%), glucose recovery rate.

**Table 2:** The analysis results of XRD and BET.

	CrI (%)	Cel (%)	CrI/Cel	Pore Volume (cc/g)	Surface Area (m^2^/g)	Pore Width (nm)
RM	58.77	42.90	1.37	0.02	0.81	2.77
PM	69.16	58.80	1.18	0.02	1.07	2.84

Footnots: CrI, crystallinity index; Cel, cellulose content.

**Table 3:** Optimization of a combined enzyme-additive system for low-loading saccharification of reed residue.

Code	ΒGL (FBG/g)	SL (mg/g)	BSA (mg/g)	GC_48 h	GY_48 h	GC_72 h	GY_72 h
1	6	40	40	31.40 ± 0.38	59.88 ± 0.72	36.91 ± 0.52	70.37 ± 1.00
2	6	60	60	33.33 ± 0.44	63.56 ± 0.84	38.17 ± 0.24	72.78 ± 0.45
3	6	80	80	29.10 ± 0.28	55.50 ± 0.53	35.30 ± 0.42	67.31 ± 0.79
4	9	40	60	38.50 ± 0.54	73.41 ± 1.03	41.72 ± 0.37	79.55 ± 0.70
5	9	60	80	37.16 ± 0.39	70.86 ± 0.75	40.63 ± 0.27	77.48 ± 0.51
6	9	80	40	37.47 ± 0.32	71.44 ± 0.60	39.45 ± 0.34	75.22 ± 0.64
7	12	40	80	38.60 ± 0.32	73.61 ± 0.61	41.96 ± 0.36	80.00 ± 0.69
8	12	60	40	39.55 ± 0.34	75.41 ± 0.65	42.78 ± 0.38	81.58 ± 0.73
9	12	80	60	39.03 ± 0.40	74.42 ± 0.76	41.38 ± 0.35	78.91 ± 0.67
Average 1	70.15	75.72	76.64				
Average 2	77.42	77.08	77.28				
Average 3	80.16	74.93	73.81				
Range	10.01	2.15	3.47				

Footnotes: BGL, β—glucosidase; SL, sophorolipid; BSA, bovine serum albumin; GC, glucose concentration; GY, glucose yield. The results of the orthogonal experiment analysis are based on the data of GY_72 h.

**Table 4:** The effect of feeding modes on the high-solids enzymatic hydrolysis (25%, *w*/*v*).

Mode	Feeding Strategies					GC_72 h	GY_72 h	GC_96 h	GY_96 h	GC_120 h	GY_120 h
	0 h	5 h	10 h	15 h	20 h						
1	25%					54.63 ± 0.46	33.33 ± 0.28	73.80 ± 0.93	45.03 ± 0.57	81.36 ± 0.87	49.65 ± 0.53
2	10%	8%	7%			66.47 ± 0.35	40.56 ± 0.21	91.15 ± 0.74	55.62 ± 0.45	99.40 ± 0.73	60.65 ± 0.45
3	10%	6%	4%	3%	2%	80.51 ± 0.48	49.13 ± 0.29	111.33 ± 0.91	67.93 ± 0.56	121.36 ± 0.99	74.05 ± 0.60
4	9%	7%	5%	4%		75.64 ± 0.40	46.16 ± 0.24	104.80 ± 1.01	63.95 ± 0.61	114.46 ± 0.84	69.84 ± 0.51
5	9%	6%	4%	3%	3%	83.86 ± 0.38	51.17 ± 0.23	114.84 ± 0.84	70.07 ± 0.51	126.30 ±1.15	77.07 ± 0.70
6	8%	7%	6%	4%		72.77 ± 0.77	44.40 ± 0.47	99.98 ± 0.86	61.00 ± 0.53	109.53 ± 1.06	66.83 ± 0.65
7	8%	6%	4%	4%	3%	86.21 ± 0.52	52.60 ± 0.32	117.56 ± 0.32	71.74 ± 0.19	128.71 ± 1.71	78.54 ± 1.04
8	7%	6%	5%	4%	3%	83.37 ± 0.40	50.87 ± 0.25	113.29 ± 0.76	69.13 ± 0.46	124.09± 0.89	75.72 ± 0.54
9	6%	6%	6%	4%	3%	82.02 ±0.45	50.05 ± 0.28	113.74 ± 1.10	69.40 ± 0.67	124.07 ± 0.95	75.71 ± 0.58
10	5%	5%	5%	5%	5%	79.23 ± 0.47	48.35 ± 0.28	109.96 ± 0.94	67.10 ± 0.57	119.68 ± 0.79	73.03 ± 0.48

**Table 5:** Comparison of pretreatment conditions and enzymatic hydrolysis performance for reed straw with previously reported strategies.

Pretreatment Strategy	Pretreatment Conditions	Cellulase Loading	Substrate Content	Hydrolysis Time	Glucose Yield	Ref
Hydrothermal (HTP)	180 °C, 120 min	10 FPU/g substrate	2%	72 h	87.1%	[32]
6% (*w*/*v*) Citric acid (CA)	150 °C, 60 min				63.4%	
6% CA + 100 mg/g Tween 80	150 °C, 60 min				84.2%	
Ethanol-assisted lactic acid	175 °C, 30 min	20 FPU/g substrate	2%	48 h	98.80%	[33]
DES/H_2_O (80:20) (ChCl-succinic acid-glycerol)	120 °C, 4 h	20 FPU/g substrate	2%	72 h	87.9%	[34]
DES/H_2_O (80:20) + Tween 80	120 °C, 4 h				93.1%	
20% Na_2_SO_3_ + 25% ethanolamine	170 °C, 40 min	20 FPU/g substrate	2%	48 h	77.52%	[35]
Salicylic acid-EG/H_2_O	155.10 °C, 60 min	20 FPU/g substrate	2%	48 h	94.75%	[36]
LHW–NH_3_·H_2_O/O_2_ + PEG 6000	124 °C, 1.2 MPa, 120 min	9 FPU/g substrate	2%	72 h	97.13%	[37]
**This work** (Microwave–alkali (1.5% NaOH) + ultrasound–H_2_O_2_ cascade (ambient pressure))	500 W, 15 min + 500 W, 15 min	12 FPU/g substrate	8%	72 h	82.32%	-
			25%	120 h	78.54%	

## Data Availability

The data supporting the findings of this study are included in the article. The raw datasets generated during the study are available from the corresponding author on reasonable request.
